# Letter by Mahajan Regarding Article, "A Narrow QRS Complex Tachycardia With Apparently Concentric Retrograde Atrial Activation Sequence"

**Published:** 2009-07-01

**Authors:** Rajiv Mahajan

**Affiliations:** Assistant Professor, Department of Cardiology; PGIMER; Chandigarh, India.

**Keywords:** Supraventricular tachycardia, accessory pathway, catheter ablation

Arias et al in the article 'A narrow QRS complex tachycardia with an apparently concentric retrograde atrial activation sequence' describe a case with spontaneous intra atrial block along the mitral isthmus to explain the change in atrial activation [1]. This phenomenon has been described during radiofrequency ablation while ablating along the lateral mitral annulus for a left free wall pathway [2-4]. Mitral isthmus block is very difficult to achieve even after repeated RF lesions [5]. We feel there is a simpler explanation to their finding. We have two points to make. First, in figure 1 CS 3-4 has a good A but hardly any V. Authors themselves have admitted to difficulty in advancing the  CS catheter. We strongly feel  that CS 34  was  more into atrium and not in the CS. This could have resulted in the mistaken  assumption of concentric activation. Advancing the catheter further into the CS would have confirmed this. Secondly, a change in activation can often be due to the presence of another tachycardia. To ascribe the change in activation to a mitral isthmus block, we must demonstrate  that the tachycardia cycle length and VA in the  HIS remained the same. Also, only if the concentric activation is persisting at the time of ablation, the final successful ablating site at the lateral mitral isthmus having the earliest A confirms that the block at the mitral isthmus was the cause of concentric activation. Further more advancing the CS catheter further into the CS during the time of apparent concentric activation would have confirmed the mechanism. If the CS 3-4 was out of the CS, the deeper insertion would have made the activation eccentric. If spontaneous isthmus block was actually present, double potentials along the line of block with sudden change in activation beyond it would have confirmed.
